# Evaluation of Virulence Factors, Antibiotic Resistance, and Biofilm Formation of *Escherichia coli* Isolated from Milk and Dairy Products in Isfahan, Iran

**DOI:** 10.3390/foods11070960

**Published:** 2022-03-26

**Authors:** Arghavan Madani, Zahra Esfandiari, Parisa Shoaei, Behrooz Ataei

**Affiliations:** 1 Department of Food Science and Technology, Food Security Research Center, School of Nutrition and Food Science, P. O. Box: 81746-73461, Isfahan University of Medical Sciences, Isfahan, Iran; arghavanmi@nutr.mui.ac.ir; 2 Nosocomial Infection Research Center, P. O. Box: 81746-73461, Isfahan University of Medical Sciences, Isfahan, Iran; shoaei@idrc.mui.ac.ir (P.S.); ataei@mui.ac.ir (B.A.); 3 Infectious Diseases and Tropical Medicine Research Center, P. O. Box: 81746-73461, Isfahan University of Medical Sciences, Isfahan, Iran

**Keywords:** diarrheagenic *E. coli*, antibiotic resistance, biofilm, milk, dairy products, Iran

## Abstract

Diarrheagenic *E. coli* (DEC) strains are important causes of gastrointestinal diseases worldwide, especially in developing countries. This study aimed to investigate the presence, antibiotic resistance, and potential biofilm formation in dairy products in Isfahan, Iran. A total of 200 samples, including traditional and pasteurized dairy products, were analyzed. In 200 samples, 54 *E. coli* isolates, including (48/110) and (6/90) positive samples of traditional and pasteurized dairy products, were detected. Furthermore, pathogenic strains were isolated from 30% of traditional dairy products and 5.55% of pasteurized dairy products. Most isolates were classified as enteropathogenic *E. coli* (EPEC). Moreover, antibiotic resistance was evaluated using the disk diffusion method for pathogenic *E. coli*. Overall, 73.68% of contaminated samples by pathogenic strains were resistant to at least one antibiotic. The highest resistance was observed against streptomycin (57.9%), followed by tetracycline (50%). Additionally, all isolates were sensitive to amikacin. For evaluating biofilm formation, the violet crystal assay was applied on a polystyrene microplate well for pathogenic isolates. In total, 68.42% of isolates were able to form biofilms. The presence of *E. coli* in dairy products indicates potential health risks for Iranian consumers. Serious measures are needed to control and prevent the spread of this pathogen.

## 1. Introduction

Food-borne diseases are a major public health concern around the world [1]. The rate of food-borne diseases is estimated at 30% in developed countries, and up to 2 million deaths are reported each year in developing countries [2]. *Escherichia coli* (*E. coli*) is among the 31 major pathogens that cause food-borne diseases, with adverse effects on the individual’s health [3]. This bacterium is a Gram-negative bacillus, which belongs to the Enterobacteriaceae family and the genus *Escherichia*. The lower intestine of warm-blooded humans and animals have a low abundance of *E. coli* (0.1% of the entire gut microbiome), which is mostly without harmful effects [4,5,6]. However, there are *E. coli* groups with virulence factors that can lead to diarrheal diseases in healthy humans [7]. Therefore, *E. coli* as an indicator of fecal contamination has been used to evaluate the hygienic quality of foodstuffs for nearly a century [8,9].

The most well-studied diarrheagenic *E. coli* (DEC) pathotypes include enteropathogenic *E. coli* (EPEC), enteroaggregative *E. coli* (EAEC), enterotoxigenic *E. coli* (ETEC), enteroinvasive *E. coli* (EIEC), enterohemorrhagic (Shiga toxin-producing) *E. coli* (EHEC/STEC) [10]. The DEC contain adhesive factors, which allow them to settle beyond the natural environment of the large intestine as hair-like fimbriae [6]. Detecting DEC strains involves the identification of phenotypic features and encoded genes [5]. EPEC, which is a major cause of potentially fatal diarrhea in infants, contains attaching and effacing (*eaeA*) and bundle-forming pilus A (*bfpA*) genes, which play a role in the attachment of bacteria to intestinal epithelial cells and lead to significant cytoskeletal alterations and polymerized actin accumulation under the adherent bacteria [5,8]. Isolates with *bfpA* and *eaeA* are known as typical EPEC (tEPEC), while negative bfpA isolates are classified as atypical EPEC (aEPEC) [5].

Among the recognized DEC, ETEC is a major cause of diarrhea in travelers and young children every year, particularly in developing countries [11,12]. The ETEC is involved in pathogenesis by producing heat-labile (lt) and heat-stable (st) enterotoxins [8]. The EIEC can attack colonic epithelial cells and replicate within epithelial cells and macrophages [8]. It is phenotypically and genotypically similar to Shigella and causes invasive and dysenteric diarrhea [5,13]. EAEC infectivity appears to be accomplished by the colonization of the small and large bowel mucosal surfaces and the elaboration of enterotoxins [14]. It is known as the cause of acute and persistent diarrhea [5]. STECs produce cytotoxins called Shiga Toxin 1 (stx1) and Shiga Toxin 2 (stx2). In addition to causing diarrhea, this strain can lead to HUS, which is associated with acute renal failure and anemia [6]. The most common serotype associated with the outbreak of EHEC in the food industries is the serotype *E. coli* O157:H7, which is a cause of major concern [4].

Foods of animal origin are major sources of food-borne diseases, which often lead to gastrointestinal disorders [2]. Milk and dairy products may be contaminated with different microorganisms, which are associated with farm animal diseases due to poor sanitation, inappropriate storage conditions, and inexperienced workers [4,15]. Obviously, the safety of milk-borne pathogenic bacteria is important for public health. *E. coli* is an important human bacterial pathogen that can be transferred to milk and dairy products [4]. However, the lack of data on contamination with *E. coli* in milk and dairy products in developing countries, such as Iran, has posed serious challenges to disease surveillance and risk assessment [16].

The dairy industry of Iran is one of the largest dairy industries in the Middle East. The dairy products per capita usage was estimated at 95 kg in 2003, which is about 10 kg more than the rate reported in 2002, whereas in 2014, it reduced to 60 kg [17]. Overall, the consumption of traditional unprocessed raw milk-based products is popular in Iran for cultural reasons, as well as the accepted health benefits [18]. On the other hand, the consumption of traditional milk products and raw milk leads to the outbreak of food-borne pathogens [19].

Although dairy products may be harmful to consumers due to the potential presence of *E. coli*, another factor that could exacerbate the problem is the widespread use of antibiotics in the livestock industry to prevent disease and increase growth. The irrational and uncontrolled use of antimicrobials in human and veterinary medicine is associated with the antimicrobial resistance spread in bacteria, which has become a main health problem worldwide [20,21]. Genetic factors, such as horizontal gene transfer and clonal development of resistant isolates, contribute to the antibiotic resistance of bacteria [22]. Overall, the development of antimicrobial resistance in the colonizing pathogens of animals leads to the distribution and emergence of resistant *E. coli*, which can be transferred to humans through consumption or contact with infected milk or milk products [23]. Antimicrobial-resistant infections cause the death of almost 700,000 people annually around the world, and this rate is predicted to reach 10 million by 2050 [24]. A noticeable increase in antimicrobial resistance in Gram-negative bacteria, including *E. coli* in Iran over the years [21], highlights the importance of investigating the antimicrobial resistance of *E. coli* in milk and dairy products.

Biofilms are responsible for many food-borne pathogen outbreaks. Food industries face the challenge of microbial biofilm formation by *E. coli*, which can have negative implications for food safety globally [25,26]. Around 80% of microbial infections that occur in the United States are caused by food contamination with biofilm [27]. They are formed by a community of microorganisms, which grow and bind to each other on surfaces, which leads to the adhesion of cells by producing extracellular polymeric substances [28]. The process of biofilm formation by *E. coli* occurs within only 2 hours, and biofilms can persist in the environment of food processing plants for up to 10 years despite regular cleaning and sanitation [16]. Overall, the formation of biofilms by E. coli due to the production of extracellular polymer matrix and protection of microorganisms against disinfectant compounds can increase the possibility of pathogenicity [22].

Although the presence of DEC strains in various food products has been investigated in several countries, most studies in Iran have only examined milk and dairy products for the existence of pathogenic *E. coli* and have emphasized the identification of the EHEC serotypes such as *E. coli* O157:H7. However, the prevalence of DEC strains in Iranian dairy products is not well-established. Therefore, the present study aims at determining the phenotypic and molecular features of *E. coli* in raw milk, traditional dairy products, and pasteurized dairy products, collected from various markets in Isfahan, Iran, and to identify pathogenic *E. coli* in terms of virulence groups and antibiotic resistance profiles according to their origin and possibility of biofilm generation. Overall, our results can be used in addition to the available information on DEC in milk and dairy products.

## 2. Materials and Methods

### 2.1. Sampling

The present study was performed using the multistage sampling method in Isfahan, Iran, which is a subtropical city with arid summers (mean temperature, 16 °C; mean rainfall, 120 mm; altitude, 1555 m; longitude, 51°30 E; latitude, 32°31 N) and 15 municipal districts. The samples were distributed in bulk (traditional products) and packaged (industrialized and pasteurized products approved and certified by the Iranian Ministry of Health) in two groups of supply centers, including supermarkets and bulk grocery stores. The dairy products were classified into 9 groups, including boiled milk, cheese, kashk, yogurt, ice cream, butter, pizza cheese, cream, and doogh (yogurt drink), and 10 samples were collected from each product. In total, 90 samples of each group of traditional (raw-based milk) and industrialized products were prepared. In addition, 20 samples of raw milk, which were distributed only in bulk stores, were purchased. Raw milk samples are also classified in the traditional dairy samples. Overall, 110 and 90 samples of traditional and pasteurized dairy products were collected, respectively. All samples were transferred immediately to the laboratory of Infectious Diseases and Tropical Medicine Research Center in Isfahan University of Medical Sciences in sterilized plastic bags in a cold box.

### 2.2. Culture, Screening, and Biochemical Tests of E. coli

Nearly 25 g of homogenized, solid dairy specimens and 25 mL of liquid dairy and milk products were mixed with 225 mL of phosphate-buffered saline (pH = 7.2; Merck, Darmstadt, Germany), respectively, followed by shaking for 1 minute. Next, 1 mL of the homogenized solution was inoculated into EC-Broth medium (10 mL; Merck, Darmstadt, Germany) and incubated for 24 h at 44.5 °C. Following incubation, 15 µL of gas-positive tubes was streaked on MacConkey Agar (Merck, Darmstadt, Germany), and the cultures were incubated at 37 °C for 24 h. We considered the red/pink colonies suspicious and were streaked on the eosin methylene blue (EMB) agar plates (HiMedia, Mumbai, India). The presumptive *E. coli* colonies (metallic green glossy colonies with a dark or purple center) were identified by Gram staining and oxidase and catalase tests.

The confirmation of presumptive *E. coli* isolates was performed by biochemical identification (IMViC tests). All isolates were subcultured on different media, including the Sulfide Indole Motility (SIM) medium, Methyl Red/Voges-Proskauer (MR-VP) medium, Simmons Citrate agar, Lysine Indole Motility (LIM) medium, and Triple Sugar Iron (TSI) agar, for further identification and biochemical tests.

Biochemically confirmed *E. coli* isolates were selected and subcultured again on the brain-heart infusion (BHI) agar for molecular confirmation. Finally, the isolates were kept at −70 °C in trypticase soy broth with glycerol (20% *v*/*v*) for additional tests [22,29].

### 2.3. Extraction of Genomic DNA and Molecular Identification of E. coli

For DNA extraction, working cultures were prepared on BHI agar followed by incubation at 37 °C for 24 h. 3 to 5 colonies were suspended in 300 µL of lysis buffer (40 mM Tris-acetate, pH = 7.8; 20 mM sodium acetate; 1 mM EDTA; and 1% SDS) and were subjected to incubation for 20 min at 40 °C. Next, the cell pellet was resuspended in 200 µL of NaCl (5 M) solution for the removal of proteins and cell debris and inverted at least 20 times. The mixture was centrifuged at 14,000 rpm for 20 min. Then, 400 µL of absolute ethanol (96% vol/vol) and 20 µL of 3 M sodium-acetate were added to the vial. Following incubation at −20 °C for 90 min, DNA was precipitated through centrifugation at 12,000 rpm for 20 min. Then, the supernatant was dried in thermoblock while the lids of the microtubes were open, dissolved in 50 µL of deionized water, and kept at −20 °C for further use [30]. The extracted DNA templates were calculated using a NanoDrop spectrophotometer (Thermo Fisher Scientific, Waltham, MA, USA) and assessed via 1.5% agarose gel electrophoresis. Moreover, the purity of the samples was examined in the wavelengths of 260 nm and 280 nm. To detect virulence factors, 2 4-plex polymerase chain reaction (PCR) reactions were performed with multiple target gene templates to detect the strains. The primers are presented in Table S1 [31].

Each PCR tube had a total volume of 25 µL, consisting of 10 pmol/µL of each primer (1 µL) and 5 µL of extracted DNA in 12.5 µL of 2× PCR Master Mix (Amplicon, Odense, Denmark), along with 5.5 µL of nuclease-free water. The PCR protocol was as follows: denaturation at 95 °C for 5 minutes, and then, 36 cycles of 95 °C for 45 s, annealing at 50.5 °C for 45 s and at 72 °C for 45 s, and a final extension step at 72 °C for 10 min in a thermal cycler (Bio-Rad Laboratories, Hercules, California, USA). The PCR products were electrophoresed on 2% (*w*/*v*) agarose gel, consisting of Safe DNA Gel Stain (SinaClon, Tehran, Iran), and visualized under a UVIdoc transilluminator. A 50-bp DNA ladder (SinaClon, Iran) was also used as a molecular weight marker. The control strains included *E. coli* O157:H7 ATCC43895 and *E. coli* ATCC25922 (non-pathogenic). Furthermore, positive PCR samples for specific primers were confirmed using a single PCR assay [31,32].

### 2.4. Antibiotic Resistance Testing

Antibiotic resistance of all *E. coli* isolates was evaluated using the Kirby-Bauer disk diffusion in Mueller-Hinton agar plates (Gibco, Middleton, WI, USA), considering the instructions of the Clinical and Laboratory Standards Institute [33].

In the current study, bacterial suspension densities comparable to 0.5 McFarland turbidity standard were prepared. The following antibiotic discs (Oxoid^TM^, Hampshire, UK) were used: ampicillin (AM, 10 μg), amikacin (AN, 30 μg), amoxicillin/clavulanic acid (AMC, 20/10 μg), cefoxitin (FOX, 30 μg), cefotaxime (CTX, 30 μg), ceftriaxone (CRO, 30 μg), chloramphenicol (C, 30 μg), ciprofloxacin (CP, 5 μg), trimethoprim/sulfamethoxazole (SXT, 125/23.75 μg), gentamycin (GM, 10 μg), kanamycin (K, 30 μg), nalidixic acid (NA, 30), tetracycline (TE, 30 μg), and streptomycin (S, 10 μg). The inoculated plates of all pathogenic *E. coli* isolates were incubated aerobically at 37 °C for 18–24 h. Besides, *E. coli* ATCC25922 was used as a quality control strain. Finally, multidrug resistance (MDR) was considered as resistance against at least 3 or more classes of antibiotic agents [34].

### 2.5. Biofilm Formation Assay

In brief, pathogenic strains were grown in tubes, including 5 mL of Luria-Bertani (LB) broth (QueLab, Montreal, QC, Canada) and incubated overnight at 35.5 °C. Next, 1.3 µL of overnight cultures were inoculated in 96-well polystyrene flat-bottomed microplate wells (SPL Life Sciences, Pocheon, Korea), containing 130 μL of LB broth. The plates were incubated overnight at 30 °C without shaking, and the optical density (OD) of each well was determined at the absorbance of 620 nm, using a BioTek Epoch^™^ Multi-Volume Spectrophotometer System (BioTek Instruments, Winooski, VT, USA).

After removing the broth, the wells were rinsed once with sterile saline (150 μL) to remove any adherent bacteria; they were left to air-dry for 20 min. Next, all of the wells were stained with 130 μL of 1% crystal violet for 5 min. After discarding the colorant, the wells were rinsed 4 times with distilled water (180 μL) and air-dried for 1 hour. To each well, 130 μL of 96% ethanol was added to dissolve the residual stain in the biofilm matrix. The OD of stained and attached bacteria, as well as the control wells, was read at 540 nm. The extent of biofilm formation was measured based on the following formula:

BFI = (*AB* − *CW*)/*G*

where BFI represents the biofilm production index; *AB* denotes the OD_540_ of attached and stained bacteria; *CW* represents the OD_540_ of stained control wells without bacteria; and *G* is the OD_620_ of cells grown in the suspended culture. The assays were performed in triplicate [35]. A BFI value of 0.35–0.69 indicated week biofilm formation; a BFI value of 0.70–1.09 indicated moderate biofilm formation; a BFI value >1.10 indicated strong biofilm formation; and a BFI value <0.35 indicated no biofilm formation [15].

### 2.6. Statistical Analysis

Data analysis was done using SPSS 20. Fisher’s exact test and Chi-square test were employed to determine significant correlations. *p*-value less than 0.05 was regarded as significant.

## 3. Results

### 3.1. Prevalence of E. coli Isolates

Of 200 samples of dairy products tested in this study, *E. coli* was isolated from 54 (27%) samples based on their morphological, biochemical, and molecular characteristics (Table 1).

The prevalence of *E. coli* isolates was higher in traditional dairy products (48/110, 43.6%) compared to pasteurized dairy products (6/90, 6.7%) (*p* < 0.001). Among 54 positive colonies, 38 (70.4%) isolates were identified as EPEC (n= 20), ETEC (n = 7), EIEC (n = 7), and STEC (n = 4). The EPEC was the most common pathotype, as 20/54 (37%) isolates exhibited the presence of *eaeA* and *bfpA* genes. The ETEC, as well as EIEC, was detected in 7 out of 54 isolates (13%) due to the presence of related genes. The *stx2* gene for STEC was identified in 4 out of 54 isolates (7.40%). However, no EAEC isolate was found in any of the dairy products. The virulence genes in pathogenic *E. coli* isolates from traditional and pasteurized dairy products are shown in Table 2.

These virulence genes were more commonly found in raw milk (45%) (*p* < 0.05) compared to other samples. In raw milk, EPEC was the most common pathotype (30%), followed by STEC (10%) and ETEC (5%), respectively. On the other hand, in butter, cream, and pizza cheese, EPEC was the most common pathotype (20%, 15%, and 15%, respectively) (Table S2). There was no significant relationship between the virulence genes and the type of dairy samples.

### 3.2. Antibiotic Resistance Patterns of E. coli

**Figure 1:**
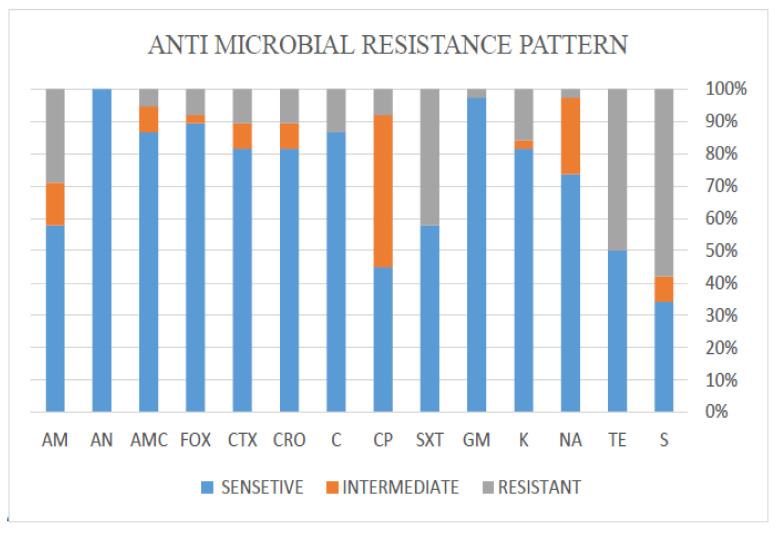
Antimicrobial resistance patterns of 38 *E. coli* strains isolated from traditional and pasteurized dairy products. AM: ampicillin, AN: amikacin, AMC: amoxicillin/clavulanic acid, FOX: cefoxitin, CTX: cefotaxime, CRO: ceftriaxone, C: chloramphenicol, CP: ciprofloxacin, SXT: trimethoprim/sulfamethoxazole, GM: gentamicin, K: kanamycin, NA: nalidixic acid, TE: tetracycline, S: streptomycin.

Out of 38 virulent *E. coli* isolates evaluated for antibiotic sensitivity, 16 (42.10%) were MDR. All MDR strains were obtained from traditional dairy products (*p* < 0.001), and 7 were detected in raw milk. The antibiotic susceptibility profile of 38 *E. coli* isolates is shown in Figure 1.

All isolates were susceptible to amikacin. Susceptibility to gentamicin, cefoxitin, amoxicillin/clavulanic acid, and chloramphenicol was estimated at 97.4%, 89.5%, 86.8%, and 86.8%, respectively. The analysis of antibiotic susceptibility revealed that more than 70% of pathogenic strains (28/38) were resistant to 1 or more tested antibiotics. More than half of the strains showed resistance to streptomycin and tetracycline. A significant difference was detected in the relationship between resistance against tetracycline and the type of samples (traditional or pasteurized products) (X^2^ = 5.76, df = 1, *p* = 0.016). Additionally, the ETEC isolates (*st* gene) were resistant to 1 or more antibiotics. Pathogenic *E. coli* strains isolated from Kashk were all resistant to streptomycin and tetracycline, pathogenic *E. coli* strains collected from ice cream were all resistant to streptomycin, and pathogenic *E. coli* strains isolated from yogurt were all resistant against tetracycline and sulfamethoxazole/trimethoprim.

### 3.3. Biofilm Formation of E. coli

All 38 pathogenic isolates were evaluated for biofilm formation. Overall, 11 (28.9%) isolates classified as strong biofilm producers, 4 (10.5%) isolates showed moderate biofilm formation, 11 (28.9%) isolates showed weak biofilm formation, and 12 (31.6%) isolates did not form any biofilms. The biofilm formation patterns of *E. coli* strains from traditional and pasteurized dairy products are summarized in Table S3. The biofilm formation of isolated pathogenic *E. coli* from different dairy products, regardless of the product group (traditional vs. pasteurized), is shown in Table S4.

Based on the results, the majority of strains, categorized as strong biofilm producers, were collected from traditional dairy products. There was a significant relationship between pathotypes and biofilm formation (*p* < 0.05). The evaluation of the antibiotic resistance patterns of antibiotics and biofilm formation showed a significant relationship between tetracycline resistance and biofilm formation in all studied products (*p* < 0.05) (Table 3).

## 4. Discussion

The prevalence of DEC is unknown in Iran, and little is known about the prevalence of these strains in milk and dairy products. Also, Iranian health authorities have not reported any statistics for this disease. Besides, no standard guideline has been developed for the control and prevention of DEC, and there are no available tests in Iran. Generally, the eating habits of Iranians differ from those of Western populations. Although several dishes have entered the Iranian diet, there are many popular traditional food recipes and products. Iran is one of the largest manufacturers of dairy products in the Middle East. The first step to convince the legislative system and the food industry to monitor the DEC strains is to investigate their prevalence rates. The present study revealed that the likelihood of infection is relatively high after the consumption of milk and dairy products, resulting in poisoning in humans. To the best of our knowledge, this is the first study on the prevalence, antibiotic resistance, and possible biofilm formation of DEC strains in traditional and pasteurized dairy products in Iran.

In this study, *E. coli* was found in 54 dairy samples (27%). Comparison of our results with some previous studies, it is indicated higher, lower, and similar prevalence rates. The contamination rate in this study was almost similar to the prevalence reported in Iran (181/600 samples; 30.16%) [36] and Ethiopia (129/380 samples; 33.9%) [37]. On the other hand, the prevalence rate observed in our study was higher than in some studies. The overall prevalence of *E. coli* has been reported in milk and dairy products in Iran (50/600 samples; 8.3%) [38] and Brazil (28/147 samples; 19.9%) [39]. Compared to the present study, higher rates have been reported in the literature for the prevalence of *E. coli* in dairy products. For example, the prevalence rate was estimated at 52.4% (11/21 samples) in China [34] and 68.7% (103/150 samples) in Iran [40]. The findings of the present study regarding the presence of *E. coli* in packaged and pasteurized dairy products revealed that the prevalence of *E. coli* in pasteurized dairy samples was 6.7% (6/90 samples), which is in line with the prevalence of pasteurized dairy products reported by Rosario et al. in 2021 (9/138, 6%) [41]. Generally, the existence of non-infectious *E. coli* in dairy products is not risky, whereas the presence of *E. coli* cells with virulence factor pathogenicity is risky [29].

In the present study, 38 out of 200 samples (19%) were contaminated with DEC. This situation was also observed in 5.5% of pasteurized dairy products (90 samples) and 30% of raw milk and traditional dairy products (110 samples). It should be noted that these isolates were characterized into different pathotypes (Table 2 and Table S2). The difference in the prevalence rates can be related to continuous monitoring of food health and safety authorities in Iran concerning the appropriate packaging and storage of industrialized food products (*p* < 0.001). Among the identified pathotypes, EPEC had the highest prevalence (20 EPEC pathotypes in 54 *E. coli* isolates; 37%). A total of 14 isolates were identified as atypical EPEC and 6 as typical EPEC (Table 2 and Table S2). Based on the results, 10% of all milk and dairy product samples showed EPEC contamination. In another study in Iran, 17 out of 206 (8.25%) raw milk samples were positive for EPEC [42]. Moreover, in a study conducted in Brazil, a prevalence of 22.1% was reported for EPEC in pasteurized milk isolates [43]. In Mexico, 13 out of 190 (6.84%) *E. coli* isolates were EPEC, which is lower than the rate measured in the current study. Also, the higher prevalence of atypical EPEC versus the typical type in the present study is consistent with the results of their study [5]. Although in our study, the prevalence of atypical EPEC was higher than typical EPEC. This situation can be attributed to the transfer of *E. coli* from livestock to dairy products or hygiene non-compliance of the personnel working in the processing environment (Table 2). Typical EPEC is the main cause of diarrhea in developing countries, and it is rarely detected in industrialized countries. The prevalence of atypical EPEC has been higher than typical strains in recent years; accordingly, they have been introduced as newly emerging strains [22,44].

In the present study, following EPEC, the highest prevalence was attributed to ETEC, with a prevalence of 13% (7 ETEC pathotypes in 54 *E. coli* isolates); the contamination rate of milk and dairy products was estimated at 3.5%. A study conducted in Italy showed that 23 out of 149 isolates (15.43%) from raw and filtered milk were ETEC [45]. However, the rate reported in the present study is higher than in some previous studies. For instance, in Iran, ETEC was not identified in any of the dairy samples (n = 102) [46]. Also, in another study conducted in Iran, only one ETEC isolate was found in 111 raw milk and 39 cheese samples [40].

In the current study, the frequency of EIEC, similar to ETEC, was estimated at 13%, and the rate of contamination was 3.5% in all examined samples. Correspondingly, a study in South Africa, investigating 46 samples of pasteurized and non-pasteurized milk that were distributed without packaging (or in bulk), reported one positive isolate of EIEC pathotype (2%) [47]. In some other studies examining the prevalence of this pathogen in food and clinical samples, the frequency was not found to be high [48]. Moreover, in 149 isolates from bulk and filtered raw milk, which were collected from different regions of Italy, 6 were (4.02%) identified as EIEC [45]. In a study in Iran, EIEC was not found in any of the food samples, including dairy products [40].

In the current study, the prevalence of STEC was 7.40% (3/54 *E. coli* isolates), and 2% of dairy samples were infected with this pathotype, which indicates the low prevalence of infection. All of these strains showed pathogenicity of the Shiga toxin 2 (*stx2*) gene as the most important clinical gene in STEC. Generally, the likelihood of developing hemolytic uremic syndrome (HUS) due to the *stx2* gene depends on STEC [19,49]. The global estimate of acute infection with STEC is 2,801,000 cases annually, including 3890 cases of HUS. In Iran, the mortality rate of STEC ranges from 19.5% to 35% in medical centers and hospitals. With a prevalence of 18%, HUS is the second leading cause of acute kidney injury (AKI) in children in Iran [50]. The findings of the present study are in line with some previous research. In a study conducted in Iran, 6 STEC strains (4%) were found in 150 raw milk and cheese samples [40]. In Italy, analysis of 95 samples showed that 2 samples of raw milk and 1 sample of mozzarella cheese were infected with STEC (3.15%) [49]. A study conducted in Mexico investigated 143 samples of dairy products and found 3 *E. coli* samples infected with STEC (2.09%) [5]. The prevalence rate reported in the present study is higher than that of some previous studies. For instance, in a study conducted in Switzerland on 796 slices of cheese from raw milk, 39 samples were positive for the *stx* gene [51]. However, the presence of STEC in dairy products is lower than that of some previous studies. In Egypt, 36 isolates of STEC were isolated from 125 (28.8%) raw milk and dairy samples; overall, 20 isolates contained both *stx1* and *stx2* genes, 10 isolates only contained *stx1* genes, and 6 isolates only contained *stx2* genes [52]. Among isolates identified in the present study, there was no EAEC pathotype; nevertheless, in some studies, very low prevalence rates have been reported for EAEC. In Mexico, 143 *E. coli* isolates were investigated, and 2 EAEC isolates (1.39%) were identified in dairy products [5]. Of 149 bulk and filtered raw milk isolates from Italian regions, 2 EAEC strains (1.34%) were found (45). In Iran, Fallah et al. (2021) examined 111 samples of raw milk and 39 cheese samples and found 4 EAEC isolates (2.66%), all of which were found in raw milk [40].

There was no significant difference in the prevalence of *E. coli* among different dairy products in the present study (Table S2). In some studies, the prevalence of *E. coli* in products such as doogh, kashk, and yogurt, has been reported to be lower. The limited presence of *E. coli* in these products can be due to the acidic pH and high temperature of the fermentation process, which are preventive factors for the presence of *E. coli* [38,46]. Therefore, the prevalence of *E. coli* in these products is commonly attributed to cross-contamination or inattention to providing an appropriate temperature in a sufficient amount of time, especially in traditional dairy production [38]. On the other hand, the presence of *E. coli* in pasteurized milk does not indicate the ability of this bacterium to survive pasteurization temperatures and may be related to the personnel’s inattention to hygienic principles after pasteurization. Factors affecting the prevention and control of *E. coli* contamination include appropriate management of livestock, milking systems, and washing systems, observance of public health guidelines by the personnel, establishment of good manufacturing principles, and use of a hazard analysis critical control points (HACCP) system in the dairy industry. Overall, these factors can be responsible for the difference between the results of the present study and other research [1].

Currently, antibiotic resistance is recognized as a serious economic, social, and community health problem worldwide [53]. The extensive use of antibiotics in the livestock industry for disease prevention has led to MDR strains in various pathogenic bacteria, causing difficulties in their eradication [16]. This issue can be the main reason for the high prevalence of antibiotic resistance in *E. coli* isolated from raw milk samples and traditional dairy products in the present study [36]. Overall, these issues suggest the importance of continuous monitoring of antibiotic resistance in food pathogens, such as *E. coli* [54]. Beta-lactam antibiotics, such as ampicillin, cefalotin, and penicillin, are among the most effective and widely used agents for the treatment of bacterial infections due to the high immunity of food products against *E. coli*. Besides, in veterinary medicine, antibiotics, such as gentamicin, tetracycline, ampicillin, streptomycin, chloramphenicol, and trimethoprim, are widely prescribed in Iran [55]. In developing countries, tetracycline, ampicillin, and sulfamethoxazole-trimethoprim are widely used due to their low cost and availability for the treatment of diarrhea. It seems that the widespread use of these antibiotics has increased the resistance of diarrhea bacteria and raised concerns among veterinarians and physicians, particularly in developing countries [56]. In this study, MDR profile was reported for 16 out of 38 (73.3%) isolates collected from milk samples and dairy products with DEC. Almost half of the identified strains showed MDR phenotypes, which is in line with reports from Asia and Africa [57,58]. Besides, all isolates from dairy products were sensitive to amikacin in the present study. In this regard, a study investigating the prevalence of *E. coli* in 280 animal, human, and food (i.e., milk, cheese, beef, chicken, and yogurt) samples, found 216 *E. coli* isolates; none of the isolates was resistant to amikacin, which is in line with the present findings. The observed lack of sensitivity may be due to the uncommon use of antibiotics in treatments [22]. In the present study, high sensitivity to cefoxitin and chloramphenicol was observed in the isolates, which is consistent with the study by Olowe et al. (2019) in the UK [22]. Generally, chloramphenicol is an antibiotic that has not been approved for animal use. The usage of this antibiotic is generally limited, explaining the low resistance of *E. coli*. In the current study, the highest antibiotic resistance of the isolates was related to streptomycin, tetracycline, and ampicillin, respectively. Similar to the present study, high resistance to tetracycline, trimethoprim-sulfamethoxazole, and ampicillin was reported [22,40,59]. In Iran, resistance to ampicillin and tetracycline was reported to be higher than the rate measured in the present study, and resistance to gentamicin was estimated at 100%, which is not consistent with the present findings [36]. There was a significant difference between dairy types (traditional and packaged) in terms of resistance to tetracycline (*p* < 0.01).

There was no significant association between antibiotic resistance and pathogenicity. In contrast, it was reported a significant association between antibiotics and pathogenicity [60]. Tetracycline is used as an animal growth promoter. Therefore, its high resistance is not unexpected in this study [61]. Additionally, a study conducted in Egypt investigated the presence of O157:H7 *E. coli* in 1600 food samples, including 800 dairy samples and 800 meat samples. Resistance to streptomycin and tetracycline was estimated at 87.1% and 80.6%, respectively, which were higher than the rates measured in the present study [62].

In the present study, observation of MDR in *E. coli* isolates may suggest the irrational use of antimicrobial agents or genetic mutations [1,7,49]. In this study, all 16 pathogenic MDR *E. coli* isolates were collected from traditional dairy products. It should be noted that raw milk and traditional dairy products are not commonly licensed and certified, and the source of production is unknown; this group of products is not adequately supervised by the health authorities in Iran. Therefore, they may be prepared from the milk of animals with mastitis, receiving high levels of antimicrobial agents to treat their disease [63]. In a study in Ethiopia, Bag et al. (2021) reported the highest resistance to amoxicillin (94.5%), followed by ampicillin (89.5%) and tetracycline (89.5%) in the milk of animals with mastitis. They also found that *E. coli* was resistant to all antimicrobial classes in farms [64]; these results suggest an association between high antibiotic resistance and mastitis, as reported in our study. Since the resistance of *E. coli* to antibiotics acts as a potential factor in animals with mastitis, it can play an important role in the resistance of *E. coli* in the breast environment, leading to the failure of treatments with antimicrobial agents [64,65]. *E. coli* is one of the main causes of mastitis in cattle, with an increasing prevalence in recent years. In the mammary glands, *E. coli* is considered to be self-limiting. Nonetheless, the possibility of relapse is high due to the continued presence of *E. coli* in the mammary glands, which may be related to the ability of *E. coli* to adhere to and invade the mammary epithelium [64]. Besides the effect of mastitis, differences in resistance to different types of antibiotics between our study and other research may be attributed to differences in the veterinarians’ prescriptions, access to antibiotics in veterinary pharmacies, and antibiotic prices in different countries [36].

Biofilm has been introduced as a frequent source of *E. coli* infection. One of the most important reasons for introducing *E. coli* as the main cause of infection worldwide is its stability and resistance to biofilm formation, which facilitates its entry into food products, causing great economic losses in the food industry, especially the dairy industry [16]. Since biofilm formation is associated with increased tolerance for stress conditions and pathogenicity, the ability of *E. coli* strains to form biofilms represents a survival strategy and allows these microorganisms to persist longer in the environment; consequently, the likelihood of food contamination is increased [7]. In the present study, 68.42% of the isolates were able to form biofilms. Overall, 28.9%, 10.5%, and 28.9% of these isolates had a high, moderate, and weak ability to form biofilms. As shown in Table S3, biofilm formation was often higher in traditional dairy products. In another similar study, biofilm formation was examined in samples isolated from meat and dairy products. The results showed that out of 32 *E. coli* isolates, 4 (12.5%), 11 (34.37%), and 15 (46.87%) isolates had a high, moderate, and weak ability to form biofilms, respectively. Moreover, in a study by de Campos et al. (2018), 4 out of 39 (10.3%) isolates of pasteurized milk were able to produce biofilms [39]. Also, in a study by Kadhum and Khudor (2021), 9 out of 11 (81.81%) *E. coli* isolates from milk contained genes responsible for biofilm formation [66]. In the present study, no significant association was found between biofilm-forming strains and antibiotic resistance (Table 3). No significant association between antimicrobial resistance phenotypes and pathogenicity genes was reported [19]. However, it was found that all MDR isolates were able to form biofilms [22].

It also reported that *E. coli* strains, which were isolated from dairy products and were both heat resistant and MDR, had the strongest biofilm formation on polystyrene [67]. The present study also confirmed the relatively high capacity of biofilm formation on polystyrene surfaces, highlighting the importance of attention to hygiene principles in the food industry. Besides, biofilm formation facilitates the survival of bacteria at different levels, including the bovine mammary glands. Therefore, biofilm-producing isolates may be related to mastitis rather than contamination after milking [39]. Several studies have shown that *E. coli* can bind to various surfaces, including food-contact surfaces (e.g., stainless steel, polyvinyl chloride, polystyrene, polypropylene, and glass) and form biofilms. Besides, it can resist chemicals, pressure, and heating procedures in the food industry [16]. Additionally, the biofilm formation of microorganisms, including *E. coli*, can be strengthened by foods that remain on surfaces, mainly due to irregular cleaning [68]. There was no significant association between the type of dairy products and the biofilm formation ability (Table S4). Binding can occur in less than 2 hours in many bacterial species, including *E. coli* [16], and biofilm is formed in less than 24 h [28,69]. Maximum biofilm formation can be achieved by increasing the contact time to 96 h [16]. In the food industry, cleaning and disinfecting solid surfaces are usually done every 8 hours. It was shown that an 8-hour interval is appropriate for binding and formation of biofilms on stainless steel surfaces and polyvinyl chloride, which is problematic for the food industry [69]. Even if regular cleaning is done, the formed biofilms can persist for up to 10 years [70]. Overall, milk and dairy products, due to the presence of nutrients and appropriate surfaces for biofilm formation, may be contaminated with various microorganisms, such as *E. coli*, which is a potential risk factor. Therefore, continuous cleaning and disinfection are of crucial importance.

## 5. Conclusions

This study provided evidence regarding the prevalence of *E. coli* in pasteurized and traditional dairy products in Iran. The presence of pathogenic and antibiotic-resistant *E. coli* strains is considered a potential risk factor for consumers of dairy products. The existence of MDR *E. coli* in dairy products is a concerning issue, which suggests the possibility of transmission to humans and can negatively affect pharmaceutical interventions. In this study, antibiotic resistance of *E. coli* strains to antibiotics, especially streptomycin and tetracycline, was observed. Antibiotic resistance was higher in traditional dairy than pasteurized. Improper use of antibiotics in the livestock industry in Iran can be responsible for these problems. The ability of *E. coli* strains to form biofilm can lead to contamination, which endangers the health of consumers. In addition, biofilm formation can enhance antibiotic resistance. In this study, a significant relationship was seen between biofilm formation and tetracycline resistance.

The higher prevalence of DEC in traditional dairy products, compared to pasteurized products, indicates the potential of these bacteria to directly or indirectly infect consumers. This difference can be related to continuous monitoring and follow-up of food hygiene and safety authorities in Iran. Besides, due to the high tendency of the population to purchase traditional dairy products, it is necessary to provide information about their further processing (i.e., heating time and temperature) because the absence of such information can increase the likelihood of human infection and poisoning. According to the present findings, appropriate hygiene measures, such as regular hygienic supervision and training of the operators and personnel involved in the production of traditional products, are necessary. In addition, increased attention of veterinarians to the prescription of drugs can prevent the expansion of antibiotic resistance genes. Moreover, regular washing and disinfection strategies of devices and equipment can reduce the possible biofilm formation of *E. coli* in milk and dairy products. Overall, based on the present findings, consumption of industrial and pasteurized dairy products is preferred to traditional dairy products. However, further studies with a larger sample size are needed to expand our knowledge.

## Figures and Tables

**Table 1:** The prevalence of *E. coli* and its virulence factors isolated from milk and dairy products.

Sample Source		No. (%) of *E. coli* Positive	No. (%) Virulence Factor Positive
Milk	boiled	1 (10)	1 (10)
	pasteurized	1 (10)	1 (10)
	raw	18 (90)	9 (45)
Cheese	traditional	5 (50)	4 (40)
	pasteurized	ND ***	ND ***
Butter	traditional	5 (50)	3 (30)
	pasteurized	2 (20)	1 (10)
Yogurt	traditional	3 (30)	2 (20)
	pasteurized	ND ***	ND ***
Kashk *	traditional	2 (20)	2 (20)
	pasteurized	ND ***	ND ***
Cream	traditional	4 (40)	4 (40)
	pasteurized	1 (10)	1 (10)
Doogh **	traditional	3 (30)	3 (30)
	pasteurized	ND ***	ND ***
Ice cream	traditional	3 (30)	2 (20)
	pasteurized	1 (10)	1 (10)
Pizza cheese	traditional	4 (40)	3 (30)
	pasteurized	1 (10)	1 (10)
Total		54 (27)	38 (19)

* Kashk: An Iranian dairy product prepared by prolonged boiling of yogurt. ** Doogh: An Iranian drink prepared by adding salt and water to yogurt. *** ND: Not Detected.

**Table 2:** Virulence genes in pathogenic *E. coli* isolates from traditional and pasteurized milk and dairy products.

Sample Source		EPEC (*bfpA*+ *eaeA*) *	EPEC (*eaeA*)	ETEC (*lt*)	ETEC (*st*)	EIEC (*ial*)	STEC (*stx2*)
Milk	boiled	ND **	1 (10)	ND	ND	ND	ND
	pasteurized	ND	ND	ND	ND	1 (10)	ND
	raw	1 (5)	5 (25)	ND	1 (5)	ND	2 (10)
Cheese	traditional	ND	1 (10)	ND	1 (10)	1 (10)	1 (10)
Butter	traditional	1 (10)	2 (20)	ND	ND	ND	ND
	pasteurized	ND	1 (10)	ND	ND	ND	ND
Yogurt	traditional	ND	ND	ND	2 (20)	ND	ND
Kashk	traditional	ND	ND	1 (10)	ND	1 (10)	ND
Cream	traditional	1 (10)	2 (20)	ND	ND	1 (10)	ND
	pasteurized	ND	ND	ND	1 (10)	ND	ND
Doogh	traditional	1 (10)	ND	ND	ND	1 (10)	1 (10)
Ice cream	traditional	ND	ND	ND	ND	2 (20)	ND
	pasteurized	1 (10)	ND	ND	ND	ND	ND
Pizza cheese	traditional	1 (10)	1 (10)	1 (10)	ND	ND	ND
	pasteurized	ND	1 (10)	ND	ND	ND	ND
Total		6 (11.1)	14 (25.9)	2 (3.7)	5 (9.2)	7 (13)	4 (7.4)

* EPEC strains can be found in two forms, namely, typical EPEC (with *eae*A and *bfp*A genes) and atypical EPEC (without *bfp*A gene). ** ND: not detected.

**Table 3:** Antibiotic resistance patterns of biofilm-producing *E. coli* strains detected in milk and dairy products.

Antibiotic Resistance in Different Degrees of Biofilm Formation				
Antibiotics	No. (%) of Strong Biofilm Formation (n = 11)	No. (%) of Moderate Biofilm Formation (n = 4)	No. (%) of Weak Biofilm Formation (n = 11)	No. (%) of Non-Biofilm Former (n = 12)
AM	2 (18.2)	1 (25)	4 (36.4)	4 (33.3)
AN	0	0	0	0
AMC	0	0	0	2 (16.7)
FOX	0	0	1 (9.1)	2 (16.7)
CTX	1 (9.1)	0	2 (18.2)	1 (8.3)
CRO	1 (9.1)	0	2 (18.2)	1 (8.3)
C	1 (9.1)	0	1 (9.1)	3 (25)
CP	0	1 (25)	2 (18.2)	0 (0)
SXT	3 (27.3)	2 (50)	8 (72.7)	3 (25)
GM	0	0	0	1 (8.3)
K	3 (27.3)	0	2 (18.2)	1 (8.3)
NA	0	0	1 (9.1)	0
TE	5 (45.5)	2 (50)	9 (81.8)	3 (25)
S	7 (63.6)	1 (25)	8 (72.7)	6 (50)
Total	23	7	40	27

AM: ampicillin, AN: amikacin, AMC: amoxicillin/clavulanic acid, FOX: cefoxitin, CTX: cefotaxime, CRO: ceftriaxone, C: chloramphenicol, CP: ciprofloxacin, SXT: trimethoprim/sulfamethoxazole, GM: gentamicin, K: kanamycin, NA: nalidixic acid, TE: tetracycline, S: streptomycin.

## Supplementary Materials

The following supporting information can be downloaded at: https://www.mdpi.com/article/10.3390/foods11070960/s1, Table S1: The PCR primers used to identify virulence genes of pathogenic *E. coli* in this study; Table S2. Distribution of virulence genes of *E. coli* strains collected from milk and various dairy products; Table S3. Biofilm formation patterns of *E. coli* isolates detected in traditional and pasteurized dairy products; Table S4. Biofilm formation in the studied dairy products.
