# Supplementary material for: Evaluation of Virulence Factors, Antibiotic Resistance, and Biofilm Formation of Escherichia coli Isolated from Milk and Dairy Products in Isfahan, Iran
Source: Foods. 2022 Mar 26;11(7):960. doi: 10.3390/foods11070960 (PMC8997477; doi:10.3390/foods11070960)
Supplement: Supplementary file 1 [file foods-11-00960-s001.zip › foods-1605767-supplementary.pdf]

Table S1. The PCR primers used to identify virulence genes of pathogenic *E. coli* in this study.

Target	gene	sequence (5′-3′)	product size (bp)
EAEC	*aafII*	CACAGGCAACTGAAATAAGTCTGG<br>ATTCCCATGATGTCAAGCACTTC	378
STEC	*stx1*	CTGGATTTAATGTCGCATAGTG<br>AGAACGCCCACTGAGATCATC	150
STEC	*stx2*	GGCACTGTCTGAAACTGCTCC<br>TCGCCAGTTATCTGACATTCTG	255
EIEC	*ial*	GGTATGATGATGATGAGTCCA<br>GGAGGCCAACAATTATTTCC	650
EPEC	*bfpA*	AATGGTGCTTGCGCTTGCTGC<br>GCCGCTTTATCCAACCTGGTA	324
EPEC	*eaeA*	GACCCGGCACAAGCATAAGC<br>CCACCTGCAGCAACAAGAGG	384
ETEC	*lt*	GGCGACAGATTATACCGTGC<br>CGGTCTCTATATTCCCTGTT	450
ETEC	*st*	ATTTTTCTTTCTGTATTGTCTT<br>CACCCGGTACAAGCAGGAT	190

Table S2. Distribution of virulence genes of *E. coli* strains collected from milk and various dairy products.

Strain	Pathotype	No. (%) of Raw milk	No. (%) of Pasteurized milk	No. (%) of traditional milk	No. (%) Of Cheese	No. (%) of Butter	No. (%) of Yogurt	No. (%) of Kashk	No. (%) of Cream	No. (%) of Doogh	No. (%) of Ice cream	No. (%) of Pizza cheese
EPEC	*Bfpa+eaeA*	1/20(5)	ND	ND	ND	1/20(5)	ND	ND	1/20(5)	1/20(5)	1/20(5)	1/20(5)
EPEC	*eaeA*	5/20(25)	ND	1/10(5)	1/20(5)	3/20(15)	ND	ND	2/20(10)	ND	ND	2/20(10)
ETEC	*st*	1/20(5)	ND	ND	1/10(5)	ND	2/20(10)	ND	1/20(5)	ND	ND	ND
ETEC	*lt*	ND	ND	ND	ND	ND	ND	1/20(5)	ND	ND	ND	1/20(5)
EIEC	*ial*	ND	1/10(5)	ND	1/20(5)	ND	ND	1/20(5)	1/20(5)	1/20(5)	2/20(10)	ND
STEC	*stx1*	ND	ND	ND	ND	ND	ND	ND	ND	ND	ND	ND
STEC	*stx2*	2/20(10)	ND	ND	1/20(5)	ND	ND	ND	ND	1/20(5)	ND	ND
EAEC	*aafii*	ND	ND	ND	ND	ND	ND	ND	ND	ND	ND	ND

Table S3. Biofilm formation patterns of *E. coli* isolates detected in traditional and pasteurized dairy products.

	Biofilm formation index (BFI)*			
Dairy type	No. (%) of Strong	No. (%) of Moderate	No. (%) of Weak	No. (%) of Non-biofilm producer
Traditional	10 (30.3)	4 (12.1)	10 (30.3)	9 (27.3)
Pasteurized	1 (20)	0	1 (20)	3 (60)

*BFI >1.10 indicates strong biofilm formation; BFI of 0.70-1.09 indicates moderate biofilm formation; BFI of 0.35-0.69 indicates weak biofilm formation; and BFI <0.35 indicates no biofilm formation.

Table S4. Biofilm formation in the studied dairy products.

	Biofilm formation*			
Dairy sample	S (%)	M (%)	W (%)	N (%)
Milk	4/11(36.36)	0/11(0)	5/11(45.4)	2/11(18)
Cheese	1/4(25)	1/4(25)	1/4(25)	1/4(25)
Butter	1/4(25)	1/4(25)	0/4(0)	2/4(50)
Yogurt	1/2(50)	1/2(50)	0/2(0)	0/2(0)
Kashk	1/2(50)	0/2(0)	0/2(0)	1/2(50)
Cream	0/5(0)	0/5(0)	2/5(40)	3/5(60)
Doogh	0/3(0)	0/3(0)	2/3(66.7)	1/3(33.3)
Ice cream	0/3(0)	0/3(0)	1/3(33.3)	2/3(66.7)
Pizza cheese	3/4(75)	1/4(25)	0/4(0)	0/4(0)

* No significant correlation was found between dairy products and biofilm formation.
